# Multi-coefficient eigenmode operation—breaking through 10°/h open-loop bias instability in wideband aluminum nitride piezoelectric BAW gyroscopes

**DOI:** 10.1038/s41378-023-00486-3

**Published:** 2023-02-21

**Authors:** Zhenming Liu, Haoran Wen, Farrokh Ayazi

**Affiliations:** 1 School of Electrical and Computer Engineering, Georgia Institute of Technology, Atlanta, GA 30308 USA; 2 StethX Microsystems Inc., Atlanta, GA 30308 USA

**Keywords:** Sensors, Electrical and electronic engineering

## Abstract

In this paper, a modification to the eigenmode operation of resonant gyroscopes is introduced. The multi-coefficient eigenmode operation can improve cross-mode isolation due to electrode misalignments and imperfections, which is one of the causes of residual quadrature errors in conventional eigenmode operations. A 1400 µm annulus aluminum nitride (AlN) on a silicon bulk acoustic wave (BAW) resonator with gyroscopic in-plane bending modes at 2.98 MHz achieves a nearly 60 dB cross-mode isolation when operated as a gyroscope using a multi-coefficient eigenmode architecture. The as-born frequency mismatches in multiple devices are compensated by physical laser trimming. The demonstrated AlN piezoelectric BAW gyroscope shows a large open-loop bandwidth of 150 Hz and a high scale factor of 9.5 nA/°/s on a test board with a vacuum chamber. The measured angle random walk is 0.145°/√h, and the bias instability is 8.6°/h, showing significant improvement compared to the previous eigenmode AlN BAW gyroscope. The results from this paper prove that with multi-coefficient eigenmode operations, piezoelectric AlN BAW gyroscopes can achieve a noise performance comparable to that of their capacitive counterpart while having the unique advantage of a large open-loop bandwidth and not requiring large DC polarization voltages.

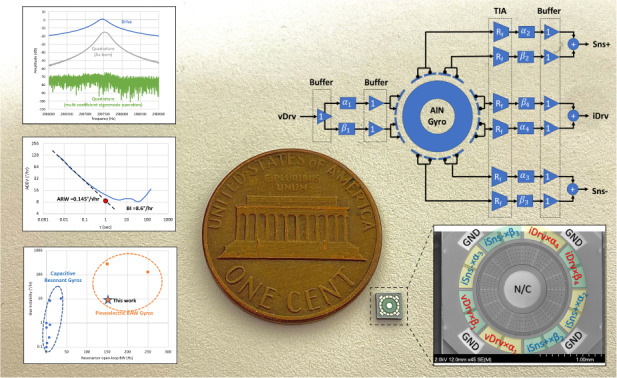

## Introduction

Gyroscopes, which are important subsystems of inertial navigation systems, have gained much attention in recent years for a multitude of applications^1,2^. The microelectromechanical system (MEMS) gyroscope, which utilizes the Coriolis effect, is adopted for chip integration due to its low SWaP-C (size, weight, power, and cost)^3–7^. A precise gyroscope with a large dynamic range has two main figures of merit: low noise and a wide operational bandwidth. Mode-matched operation of a high *Q* resonant gyroscope has proven to be efficient in improving the signal-to-noise ratio by *Q*-amplification of the rate response. However, the open-loop bandwidth for a mode-matched resonant gyroscope is inversely proportional to its *Q*, leading to a trade-off between the two figures of merit^8–13^. Although the operational bandwidth can be expanded with a force-to-rebalance operation, a complex circuit is required if the starting open-loop bandwidth is very small^14–16^. One approach to circumvent such conflict is by taking the operation frequency of the gyroscopic modes to the megahertz range using stiff bulk acoustic wave (BAW) modes and more efficient transducers^17–20^. The BAW gyroscope also shows a lower sensitivity to environmental vibrations and ambient pressure compared with lower frequency devices^21,22^.

To further increase the open-loop bandwidth for high dynamic range applications, the *Q* factor needs to be relaxed while a high signal-to-noise ratio is maintained with a stronger electromechanical coupling; this makes piezoelectric resonators with moderate *Q*s and high transduction efficiencies particularly promising to meet both requirements^23^. Compared with electrostatic resonators with nano-gap parallel plate capacitive transducers, piezoelectric resonators also show some additional advantages, including a large linear actuation range, no DC polarization voltage, and ease of fabrication^23,24^. However, the parallel plate is still the mainstream transduction mechanism for BAW gyroscopes, mostly due to the post-fabrication tuning capability with an electrostatic spring softening effect to match and align the gyroscopic modes^25–27^. Because of the lack of a tuning mechanism, especially a solution to reduce cross-mode coupling and quadrature error from mode misalignment, the noise performance of the piezoelectric BAW gyroscope has not been on par with that of state-of-the-art electrostatic devices.

Researchers have been looking for alternative tuning techniques in piezoelectric resonators over the past years. Frequency control via dynamic feedback of the displacement signal has been used to mode match piezoelectric BAW gyroscopes by shifting the resonance peak of the drive mode signal^28,29^. However, the drive mode phase noise will be reintroduced into the gyroscope output with this feedback loop and compromise the gyroscope bias stability. Physical laser trimming is another cost-efficient technique that can not only match the frequencies but also realign the mode shape without adding noise; however, the demonstrated alignment precision is not sufficient as a stand-alone solution^30–32^. Recently, an eigenmode operation was introduced for mode realignment by virtually rotating the electrodes to the actual mode directions and showed the best results to date; however, the cross-mode isolation and noise level are still not comparable to those of a capacitive BAW gyroscope^33,34^.

In this paper, the root cause of the residual quadrature error in the eigenmode operation is investigated, and the reason for the geometrical mismatch between the mode shapes and the electrodes is obtained. A modification is made based on the conventional eigenmode operation by introducing multiple coefficients that separately align the excitation and sense electrode to the modal directions. The new method, called the multi-coefficient eigenmode operation, is implemented on an annulus AlN-on-Si resonator with dimensions very similar to those of the conventional eigenmode device^34^ and shows significant improvement with regard to quadrature cancelation. Mode matching is accomplished with laser ablation trimming to bring the frequency of the two modes within −3 dB bandwidth. The characterized AlN BAW gyroscope shows a noise performance very close to the capacitive counterparts of similar size^35^. In addition, the as-born and post-trim frequency response data of several AlN BAW gyroscopes batch-fabricated on an 8-inch wafer are provided, showing good repeatability and a promising platform for low noise, wide bandwidth MEMS gyroscopes.

## Results and discussion

### Theory of eigenmode operation

The theory of the eigenmode operation has been previously outlined and explained^33,34^. In summary, a gyroscopic system can be modeled as a 2-degree-of-freedom (2DOF) system in a generalized coordinate {*q*}:

1 $$\left[ M \right]\left\{ {\ddot q} \right\} + \left[ C \right]\left\{ {\dot q} \right\} + \left[ K \right]\left\{ q \right\} = \left\{ Q \right\}$$

where [*M*], [*C*], and [*K*] are the mass, damping, and stiffness matrices, respectively. Moreover, {*Q*} is the actuation force in vector form, which is typically aligned with the primary axis of {*q*}. Notably, the damping matrix is mostly linearly proportional to [*M*] and [*K*]; moreover, the damping value is negligible for a resonator with moderate to high *Q*, such that the damping terms can often be ignored.

Due to the misalignment between {*q*} and the modal coordinate {*η*}, the off-diagonal stiffness terms in [*K*] couple energy between two modes. The transfer between {*q*} and {*η*} is:

2 $$\left\{ q \right\} = \left[ {{\Phi }} \right]\left\{ \eta \right\}$$

where [Φ] is the mass-normalized eigenvector of the 2DOF system. Ignoring damping, Eq. (1) can be rewritten in modal coordinates as:

3 $$\left[ M \right]\left[ {{\Phi }} \right]\left\{ {\ddot \eta } \right\} + \left[ K \right]\left[ {{\Phi }} \right]\left\{ \eta \right\} = \left\{ Q \right\}$$

Taking advantage of the mode orthogonality, the following can be obtained:

4 $$\left[ {{\Phi }} \right]^T\left[ M \right]\left[ {{\Phi }} \right] = \left[ I \right]$$

5 $$\left[ {{\Phi }} \right]^T\left[ K \right]\left[ {{\Phi }} \right] = \left[ \lambda \right]$$

where [*I*] is the identity matrix, and [*λ*] is a diagonal matrix with eigenvalue solutions. Therefore, the two modes can be decoupled by applying a transfer matrix [Φ]^*T*^ to Eq. (3):

6 $$\left\{ {\ddot \eta } \right\} + \left[ \lambda \right]\left\{ \eta \right\} = \left[ {{\Phi }} \right]^T\left\{ Q \right\}$$

**Fig. 1:**
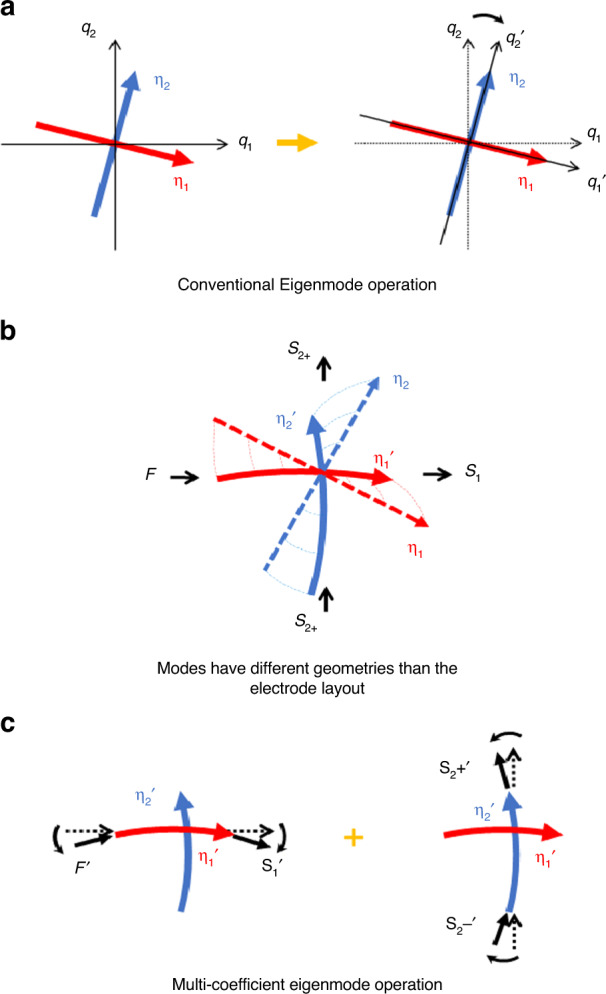
The schematic representation of conventional and multi-coefficient eigenmode operation. **a** Illustration of the modal coordinate {*η*} for a pair of orthogonal gyroscopic modes. The modal coordinate is orthogonal and has an angular offset from the general coordinate {*q*} to which the drive/readout direction are aligned (left). Such a resonator can be realigned in one rotation of the general coordinate {*q'*} and all virtual electrodes together, using the conventional eigenmode operation (right). **b** However, for nonideal resonators with mode shapes misaligned from the electrodes layout, despite the modal coordinate {*η*} still being orthogonal, the observed mode pattern {*η'*} in the general coordinates will have an arbitrary shape. Each electrode and as such, the driving direction *F*, mode 1 readout *S*_*1*_, and mode 2 differential readout S_2±_, are potentially misaligned from the actual mode shape by a different angle. **c** Complete mode decoupling in such resonators needs to be divided into separate tasks of aligning each electrode to its corresponding modal axis. The left plot aligns the *F*′ and *S*_*1*_′ direction with Mode 1, and the right plot aligns the *S*_*2±*_‘ direction with Mode 2. Since the alignment angles could be different, multiple rotation coefficients are necessary

Figure 1a demonstrates the principle of the eigenmode operation, where the generalized coordinate and the actuation/sensing direction are rotated to align with the modal coordinate. To apply such rotations to a physical BAW gyroscope, each actuation and readout electrode can be divided into pairs spanning the spatial projection of the electrode coordinate. The total driving force or readout signal is equal to the superposition of the voltage vectors in each eigenmode operation electrode. By applying the weighting coefficients *α* and *β* to change the magnitude of these two vectors, their superposition can be virtually rotated and thus aligned to the target direction. In practice, the sums *α* and *β* are kept at 1, so the total magnitude is constant.

### Compensation of geometrical mismatches between the mode shapes and electrodes

Complete mode decoupling can be divided into the following two separate tasks. The first task is only actuating the drive mode while maintaining a silent sense mode, which is done by applying the excitation force at the anti-node of the drive mode. The second task is only obtaining a readout of the signal from the sense mode while rejecting all drive mode signal, which is done by placing the sense mode readout at the anti-node of the sense mode. Because any two arbitrary modes are always orthogonal to each other with respect to their mass and stiffness matrix, as described in Eqs. (4) and (5), complete mode decoupling can be achieved in theory using the eigenmode operation to coincide with the general axis to its modal coordinate when the electrode layout is also aligned along the general axis. However, due to non-idealities, the spans of the electrodes may differ from the angle between the node and the anti-node of each mode. The electrode placement assumes an ideal mode shape and strain pattern; however, in reality, factors such as fabrication imperfections and tuning will alter the stiffness distribution and boundary conditions, causing the actual mode shape/strain distribution to slightly deviate from the ideal case and create different small misalignments within each electrode, as shown in Fig. 1b. A simulated non-ideal gyroscopic resonator will be shown in a later section as an example to demonstrate the geometrical mismatch between the electrode and mode shape in the space domain.

### Multi-coefficient eigenmode operation

Due to the different mode alignments in each electrode location, when all electrodes follow the same rotation in the general axis, the two separate tasks in complete mode decoupling cannot be accomplished at the same time. Instead, each drive/readout electrode must separately fit to the actual mode shape to align the force and readout signal to the corresponding node and anti-node. Figure 1c shows the rotation of each electrode direction with a different angle and the accomplishment of complete mode decoupling. The rotation can be achieved in the same way as conventional eigenmode operations: spanning the electrode pairs along the generalized electrode coordinate and applying weighting coefficients. Because the alignment angles are independent of each other due to arbitrary non-ideal modes, each electrode pair requires a unique coefficient to precisely decouple the two gyroscopic modes.

### AlN-on-Si annulus BAW gyroscope

**Fig. 2:**
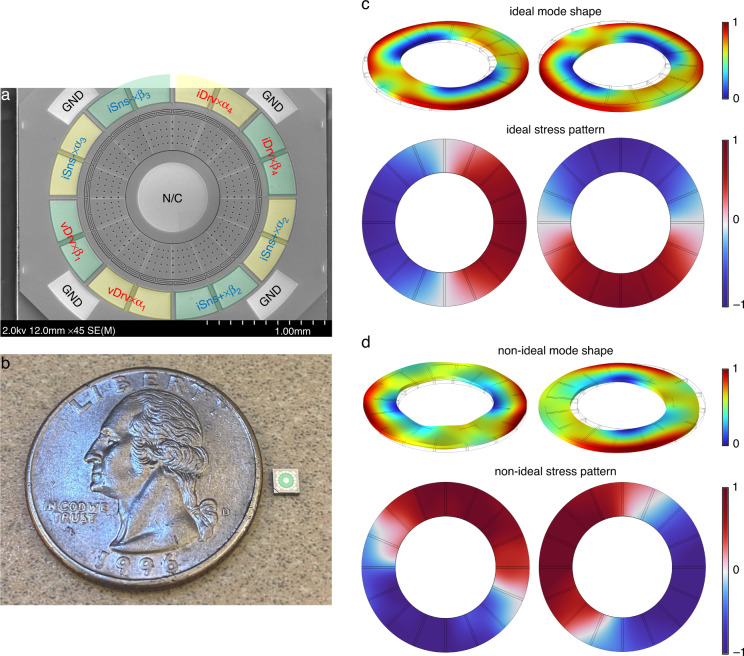
The AlN-on-Si annulus gyroscope design. **a** SEM view of a fabricated AlN-on-Si annulus resonator with 16 electrodes, each 2 adjacent electrodes are connected to the same signal. vDrv and iDrv are the electrodes for drive mode actuation and current output, iSns± are the sense mode differential output electrode. *α*_i_ and *β*_i_ are the eigenmode operation coefficients, where *β*_i_ = 1− *α*_i_ . The center pad is isolated from the device and has no electrical connection. **b** A 2.5 mm × 2.5 mm AlN gyroscope die is placed next to a US quarter coin to compare the sizes. **c** The normalized ideal mode shape and stress pattern of the fabricated resonator operating in a pair of gyroscopic in-plane bending mode. The two modes have identical stress patterns that are spatially 90° apart from each other. **d** By manually defining a nonuniform mass density to simulate the process variation, the two modes have some out-of-plane motion with different extension, changing their mode shape displacement pattern. The stress patterns are no longer symmetric and do not follow the shape of the designed electrode. This nonideal resonator is later realigned using multi-coefficient eigenmode operations in the simulations

To demonstrate the multi-coefficient eigenmode operation, we fabricated a batch of piezoelectric BAW gyroscopes with a very similar design to use for conventional eigenmode operations^34^. Each gyroscope has an outer radius of 700 µm and an inner radius of 400 µm, operating in degenerate in-plane bending modes at 2.98 MHz. To ensure that the location of the physical electrodes is closer to the mode center, we used a total of 16 electrodes uniformly distributed along the resonator, and each adjacent 4-electrode group was divided into a pair of eigenmode operation electrodes. Before using the multi-coefficient eigenmode operation to virtually fit the mode shape, we can pre-align the resonator by selectively assigning electrodes. If the as-born misalignment angle with respect to the standard electrode configuration is larger than 11.25° (span of half an electrode pad), we shift all wire-bond connections by one or two pads to physically rotate the electrodes by an increment of 22.5° until the minimum quadrature error is measured. The side-supported annulus resonator is composed of 60 µm doped (100) monocrystalline silicon with a 1 µm aluminum nitride (AlN) piezoelectric layer on top. The doped (100) silicon acts as the bottom electrode with a resistivity of <0.01 Ω cm, while the top electrode is 300-nm-thick polycrystalline silicon (poly-Si). The pads are covered with an aluminum copper film for wire bonding. Figure 2a shows a scanning electron microscope (SEM) top view of a fabricated device with the electrodes highlighted to show the electrical interfacing. Additional device SEM views as well as the fabrication process flow can be found in the supplemental information. Figure 2b compares the size of one device die to a US quarter coin.

The finite element analysis (FEA)-simulated mode shape and corresponding stress pattern for an ideal resonator are shown in Fig. 2c. Notably, since the input/output signal of a piezoelectric resonator represents stress instead of displacement, the modal coordinate and electrode configuration are based on the stress pattern. The ideal modes are 90° apart from each other along the plane normal to the rotation axis, corresponding to the electrode configuration. However, once a non-symmetric process variation, such as a local defect, is included, the two in-plane bending modes start to have some out-of-plane motion, and the angle between the stress nodes is different from the electrode span, as shown in Fig. 2d. Therefore, to realign a resonator with the actual “as-fabricated” electrode layout, each electrode needs to be aligned to the target node/anti-node separately. The control of each individual virtual electrode is similar to conventional eigenmode operations. However, a total of 4 pairs of coefficients are necessary for complete alignment. For clarification, *α*_1_ and *β*_1_ are the weighting coefficients of the driving electrode pair vDrv; *α*_2_ and *β*_2_ and *α*_3_ and *β*_3_ represent the two differential sensing electrode pairs iSns+ and iSns-, respectively; and *α*_4_ and *β*_4_ correspond to the drive mode output electrode pair iDrv. All α_i_, *β*_i_ pairs have a sum equal to 1 to maintain a constant total magnitude in each eigenmode electrode pair.

### Multi-coefficient eigenmode algorithm

**Fig. 3:**
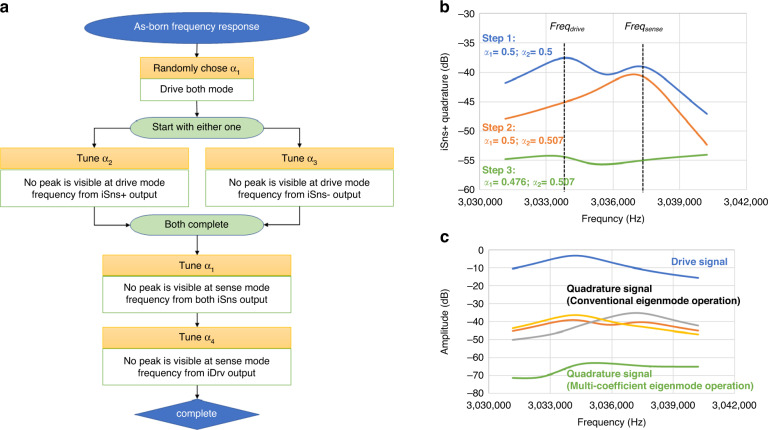
Multi-coefficient eigenmode operation algorithm and simulation results. **a** Flow chart for tunning each coefficient *α*_1~4_, after finding both mode in the frequency domain, peak of opposite mode at each driving/sensing electrode is suppressed by tuning the corresponding coefficients. **b** Example of using the frequency response of a simulated nonideal resonator (Fig. 2b) to find the coefficients for *α*_1_, the drive alignment, and *α*_2_, the readout alignment at Sns+. Step 1: purposely keep the drive mode misaligned to observed both mode in the Sns+ electrode, the frequency corresponding to each mode is labeled using the dotted line. Step 2: Adjust *α*_2_ until the peak is diminished at the drive mode frequency. Step 3: Tune *α*_1_ to eliminate the peak at sense mode frequency. **c** Compared to the conventional eigenmode operation on the device with same simulated nonideality, extra 30 dB mode isolation can be achieve using multi-coefficient eigenmode operation

The two tasks for complete mode realignment are aligning the sensing layout to the drive mode node and the driving force to the sense mode node. Because there is no apparent relationship between the location of each stress node in the non-ideal modes, the coefficients for each electrode pair are separately found, following the process shown in Fig. 3a. First, Coefficient *α*_1_ is selected arbitrarily to purposely drive the resonator with a misalignment so that the peaks for both modes are visible in the frequency domain. Then, *α*_2_ or *α*_3_ is carefully selected until the signal captured by the iSns+ or iSns− electrode is minimized at the drive mode frequency, indicating that the effective readout is now at the node of the drive mode. At this moment, remaining a high cross-coupling at the sense mode frequency is allowed since the actuation misalignment until *α*_1_ is fine-tuned to align the driving force to the node of sense mode. Finally, *α*_4_ is tuned until the drive mode electrode (iDrv) has a minimum reading at the frequency of the sense mode. In practice, after adjusting the readout coefficients *α*_2_ and *α*_3_ for iSns+ and iSns- separately, we can take the differential sense mode signal and further fine-tune *α*_1~3_ through small trials and errors until the total quadrature is minimized. Figure 3b shows a simulated frequency response example for tuning *α*_1_ and *α*_2_. The typical non-ideal frequency response with a quadrature error is reproduced in the COMSOL® FEA simulation by purposely defining a non-uniform mass density across the resonator. The mode realignment results using both conventional and multi-coefficient eigenmode operations are shown in Fig. 3c. Regarding a gyroscope with non-ideal mode shapes in contrast to the physical electrode layout/geometry, conventional eigenmode operations cannot simultaneously suppress the cross-coupling at the two frequencies due to the misalignment between the electrodes and actual mode shapes. However, in multi-coefficient eigenmode operations, each actuation/readout electrode is aligned separately, pushing forward the achieved cross-mode isolation.

### Experimental results

The experimental measurement was performed in vacuum using a mechanical pump and a sealed cap placed on top of the printed circuit board where the device was wire-bonded. Over 10 AlN-on-Si annulus resonators are characterized with a consistent center frequency at 2.98 MHz and a *Q* of approximately 10,000, corresponding to a large open-loop bandwidth of 150 Hz, which not only enlarges the gyroscopic operation bandwidth but also relaxes the frequency matching requirement. Regarding resonators with a relatively large frequency split, we use laser ablation trimming to bring the two peaks within a bandwidth of −3 dB. The as-born and post-trim frequency split and *Q* of multiple resonators fabricated on an 8″-wafer are summarized in Table 1.

**Table 1:** Summary of frequency response collected from 10 devices across an 8” wafer. First 7 devices are laser trimmed, and last 3 devices have small as-born split

Device #	Δf (Hz) As-born	Δf (Hz) Post trim	*Q*
1	371	50	10,460
2	527	63	10,297
3	285	55	10,608
4	302	58	10,667
5	294	66	10,324
6	423	54	10,218
7	131	52	10,308
8	72	N/A	10,643
9	75	N/A	10,511
10	86	N/A	10,289

The multi-coefficient eigenmode operation is implemented on resonators with small as-born or trimmed frequency splits. The coefficient for the drive mode alignment, *α*_1_, is tuned with a pair of analog voltage dividers followed by buffers with proper gains to maintain a constant total force; similarly, the readout coefficient *α*_2~4_ is tuned with the same architecture after a transimpedance amplifier (TIA) amplifies the resonator current output by a 10 kΩ feedback resistor.

**Fig. 4:**
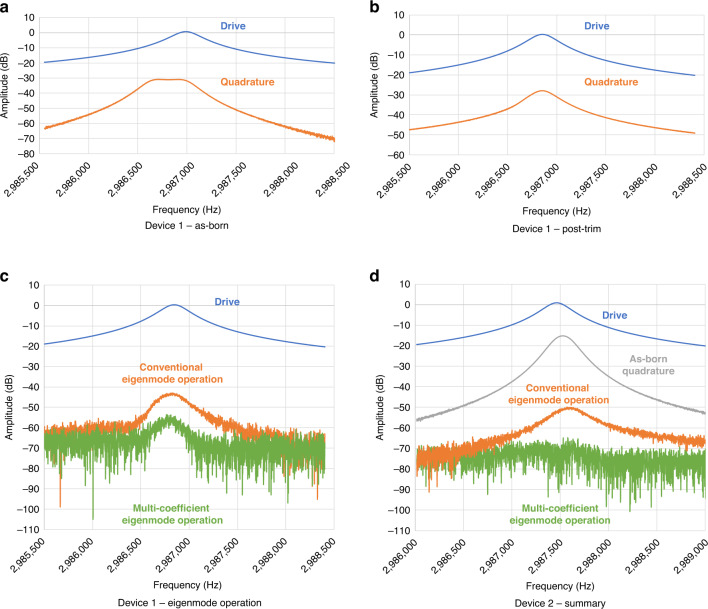
Frequency response measurement results. **a** The as-born frequency response from a device with large frequency split of 371 Hz. **b** The frequency split is reduced to 50 Hz after trimming, while the *Q* remains unchanged at 10,000. The cross-mode isolation is 28 dB. **c** Using conventional eigenmode operations, the best isolation that can be achieved is 43 dB, while for multi-coefficient eigenmode operations, an isolation of 54 dB is achieved. **d** On a different device with a small as-born frequency split of 72 Hz, the cross-mode isolation with conventional and multi-coefficient eigenmode operations are 18 dB, 51 dB, and 68 dB, respectively

The as-born frequency response of a BAW resonator along with conventional and multi-coefficient eigenmode operations are shown in Fig. 4. The mechanical trimming brings the initial 371 Hz as-born frequency split to less than 50 Hz. Conventional eigenmode operations showed a reduction of 15 dB in the cross-mode coupling, and the multi-coefficient further decouples the two modes by an additional 11 dB with a total of a nearly 60 dB mode isolation. Figure 4d shows the frequency response of another resonator with a small as-born frequency split and no trimming. Using the multi-coefficient eigenmode operation, its cross-coupling signal is completely suppressed below the noise floor, showing a mode isolation that is close to 70 dB.

**Fig. 5:**
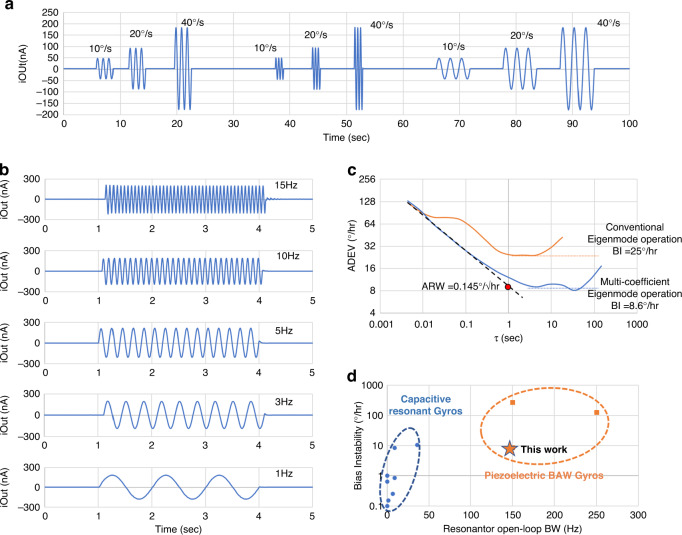
Gyroscope characterization results. **a** The gyroscope response to 10°/s, 20°/s, and 40°/s, at frequencies of 1 Hz, 2 Hz, and 0.5 Hz, respectively. The scale factor is calculated to be 9.55 nA/°/s. **b** The gyroscope response to 40°/s with frequency increasing from 1 Hz to 15 Hz, with a maximum difference output of 20 nA (2.1°/s), mostly due to the resolution of rate table. **c** The measured device has an angle random walk of 0.145°/√h, and a bias instability of 8.6°/h using multi-coefficient eigenmode operations, nearly 3× increase compared with conventional eigenmode operation for the same device with all other testing condition unchanged. **d** This work is the first-time demonstration of piezoelectric gyroscopes with noise performances at-par with capacitive gyroscopes while having a much larger open-loop bandwidth

When operated as a gyroscope, the two resonators showed very similar gyroscopic performance. The time domain waveform response with different rotation rates at multiple frequency ranges of 1, 0.5 and 2 Hz are recorded over 100 seconds (Fig. 5a). The measured scale factor is 9.55 nA/°/s with a large driving voltage of 1.9 V AC peak-to-peak. With multi-coefficient eigenmode operations, the uncompensated zero-rate output is 3.4 nA, which corresponds to a rotation bias of 0.35°/s. Because of the large open-loop bandwidth, the piezoelectric BAW gyroscope can obtain very fast rotations even without closed-loop operations. Rate responses to 20°/s sinusoidal rotation in a wide frequency range are shown in Fig. 5b. The measured gyroscope output remains unchanged in a 15 Hz bandwidth limited by the testing rate table.

The measured Allan deviation plot (Fig. 5c) shows that the AlN BAW gyroscope has an angle random walk (ARW) of 0.145°/√h and a bias instability (BI) of 8.6°/h under muti-coefficient eigenmode operations. The bias instability level occurs when the time constant τ is greater than 10 s, indicating a strong rejection of flicker noise, which is proportional to the quadrature error. The long-term noise after 100 s is mostly due to the testing setup, including the vibration from the mechanical pump and temperature drift. For comparison, when the same device is under conventional eigenmode operations with identical testing conditions, the ADEV plot is significantly affected by flicker noise, and the measured BI remains at 25°/√h. Figure 5d also compares the results shown in this work to those of other state-of-the-art devices^8,9,11,13,18,22,27,34,35^. Multi-coefficient eigenmode operations enable the piezoelectric gyroscope to have a breakthrough noise performance comparable to capacitive counterparts while maintaining a very large open-loop bandwidth.

## Material and method

### Laser trimming

Resonators with large frequency splits are mechanically trimmed with a laser-based trimming algorithm. A detailed description for guiding the trimming location and using laser ablation for mode-matching and alignment of BAW resonant gyroscopes without loading the *Q* can be found elsewhere^32^. The trimming process starts with deriving the numerical maps for the location-dependent effective stiffness or mass terms and then using laser ablation to selectively change the geometry or remove material. In this study, trimming is mostly focused on reducing the frequency split to less than a quarter bandwidth. Ideally, a zero-frequency split is desired. This requires a better resolution in the frequency measurement, given the wide bandwidth and higher precision in the laser control. We used the OPTEC WS-Flex USP system, which comprises a femtosecond laser and a computer-controlled stage with micrometer precision. This system is capable of handling 8-inch wafers, and the trimming process is scalable to the level of the wafer prior to wafer bonding occurring for vacuum encapsulation of the device.

**Fig. 6:**
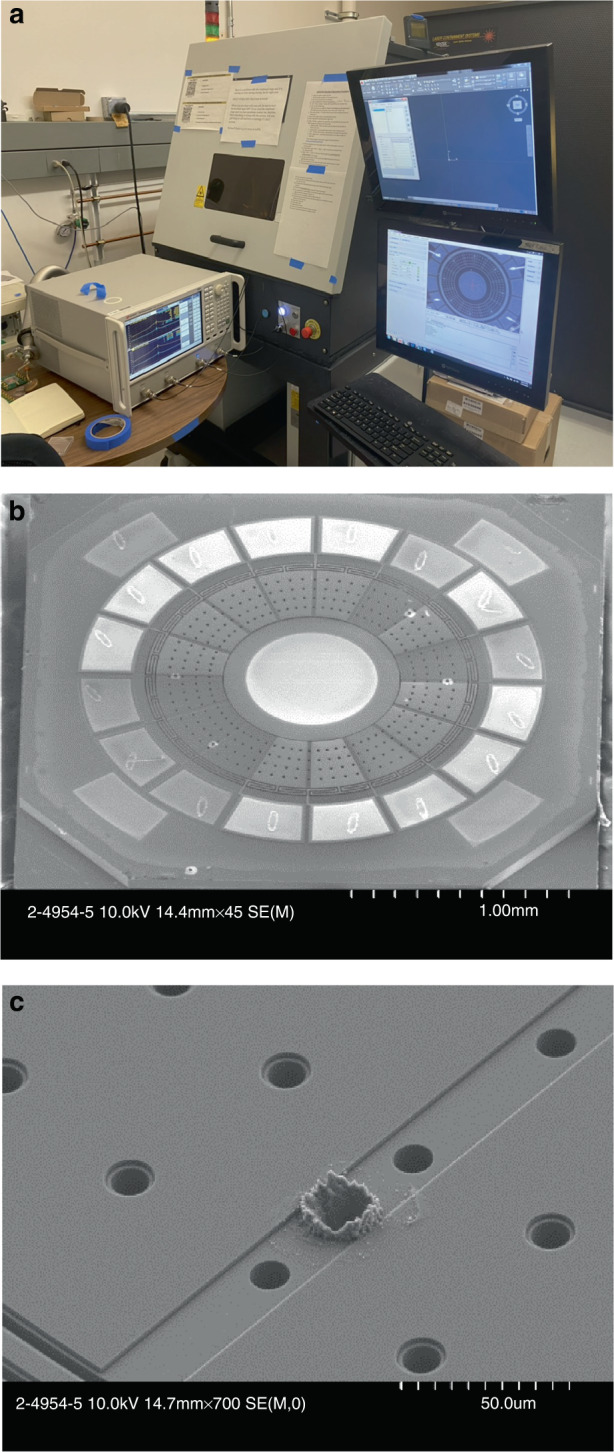
The laser trimming setup and trimming profile. **a** The OPTEC WS-Flex USP system equipped with a microscope camera for laser alignment. The resonator is wire-bonded for real-time in-air measurement during trimming. The device is transferred to vacuum for a more accurate frequency measurement between each iteration. **b** SEM image showing a post trimming device; and **c** a zoom-in picture focus on the ablation hole, in which the diameter is 15 µm, and the depth and actual geometry is challenging to precisely measure

To characterize laser trimming, we started with an iterative process. The laser power is fixed at 15 W, and the wavelength is set to 215 femtoseconds. We discretely increased the laser energy by iteratively increasing the number of pulses at the same locations guided by the map and then recorded the change in the effective stiffness versus the pulse number. Once the relationship between the laser energy and trimming effect is known, we can calculate the trimming parameters (location and laser pulse number) from the as-born frequency response and trimming map and then match the frequency in a single run. The typical required pulse number ranges from 5 to 30 depending on the as-born frequency response. The trimming setup is shown in Fig. 6a. During iterative trimming, the resonator is measured in-air with a lower *Q* of 2500 due to air damping. Since the frequency split is reduced, it is necessary to transfer the resonator into the vacuum for more accurate measurements. To avoid affecting the piezoelectric transduction, the trimming locations are limited to the exposed bottom (100) silicon device layer. Figure 6b shows the SEM image of a trimmed device with Fig. 6c zoomed in to focus on the ablation hole. Since the resonators are already perforated, additional trimming holes of comparable size do not add extra thermal elastic damping. A detailed study of *Q* preservation during laser trimming was documented in the literature^32^. In this research, the *Q* of post-trimming devices remains unchanged. Moreover, it is limited by the piezoelectric loss, as evidenced by Table 1, where the post-trimming *Q* of the trimmed devices in Rows 1 through 7 are equal to the as-born *Q* of identical untrimmed devices of Rows 8 through 10.

### Gyroscope measurement setup

**Fig. 7:**
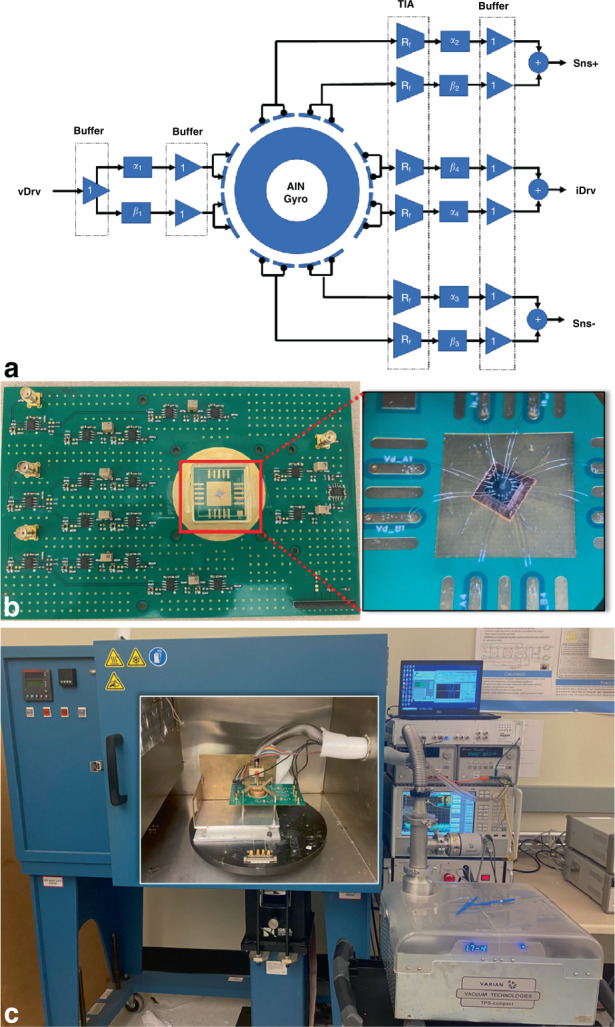
The circuitry and test setup for gyroscope measurement. **a** The schematic for multi-coefficient eigenmode operation circuitry board. **b** The printed circuit board with multi-coefficient eigenmode operation, the inset shows the device strategically selecting the wire-bond pads to minimum as-born mode misalignment. **c** The device is measured in vacuum with a mechanical pump and place inside an Ideal Aerosmith rate table. The pump is able to achieve low pressure in the order of 10^−4^ mbar but must keep constantly on due to the leak up

After laser trimming, the frequency-matched resonators are first realigned with eigenmode operations in the frequency domain following the multi-coefficient algorithm using a Keysight E5080A Network Analyzer. Then, the connection is transferred to a Zurich HF2LI lock-in amplifier to create an oscillation loop at the drive mode frequency. After properly demodulating the sense mode signal, we further fine-tune the coefficients (with very small magnitude changes) of eigenmode operations until the flicker noise is most diminished in the time domain waveform. The circuitry schematic for multi-coefficient eigenmode operations is shown in Fig. 7a. Each coefficient is controlled by a pair of voltage dividers using an analog potentiometer. A buffer with a unit gain is added after each voltage divider to maintain a high input impedance. Figure 7b shows the gyroscope wire-bonded on the multi-coefficient eigenmode operation circuit board. Figure 7c shows the whole setup: the circuit board is fixed on the rate table, with a vacuum seal cap connected to a pump. This pump is mechanically noisy, with a strong vibration coupled to the resonator through the vacuum pipeline, limiting the long-term stability.

## Conclusion

In this paper, we present AlN-on-Si piezoelectric BAW gyroscopes with bias instability below 10°/h. To the best of our knowledge, these gyroscopes have the best noise performance among similar wideband resonant gyroscopes. Compared to our previous result^34^ using a similar design, there is a 5-fold reduction in ARW and a 12-fold improvement in BI. The better noise performance results from a higher cross-mode isolation by the demonstrated multi-coefficient eigenmode operation, in which the actuation and sensing electrodes are virtually aligned with the actual mode vectors to compensate for the geometrical mismatch between the mode shape and electrodes. The given statistical data of the frequency response show consistent frequency matching with and without laser trimming, proving repeatable behavior for a piezoelectric AlN-on-Si BAW resonator as a Coriolis gyroscope. Compared with a capacitive BAW gyroscope of similar size, the demonstrated piezoelectric BAW gyroscope shows a comparable noise performance while maintaining its unique advantage of large open-loop bandwidth, lack of DC voltage, and ease of fabrication. For future work, wafer-level vacuum packaging will be provided to eliminate the external vacuum pump setup noise. A force-to-rebalance closed loop can help be implemented to further improve bias instability and long-term drift.

## Supplementary information

AlN-on-Si Resonator Gyroscope Fabrication Process
