# Supplementary material for: Multi-coefficient eigenmode operation—breaking through 10°/h open-loop bias instability in wideband aluminum nitride piezoelectric BAW gyroscopes
Source: Microsyst Nanoeng. 2023 Feb 21;9:18. doi: 10.1038/s41378-023-00486-3 (PMC9945455; doi:10.1038/s41378-023-00486-3)
Supplement: Supplementary file 1 — AlN-on-Si Resonator Gyroscope Fabrication Process [file 41378_2023_486_MOESM1_ESM.pdf]

Supplementary Information for

Multi-Coefficient Eigenmode Operation – Breaking Through 10°/hr Open-Loop Bias Instability in Wideband Aluminum Nitride Piezoelectric BAW Gyroscopes

Zhenming Liu[1] , Haoran Wen[2], and Farrokh Ayazi[1,2]

[1] School of Electrical and Computer Engineering, Georgia Institute of Technology, Atlanta, GA 30308, USA

[2] StethX Microsystems Inc., Atlanta, GA 30308, USA

Email: [1]zhenming_liu@gatech.edu; [2]haoran@stethx.com; [1,2]ayazi@gatech.edu

AlN-on-Si Resonator Gyroscope Fabrication Process

The AlN-on-Si resonator gyroscope is fabricated on 8-inch silicon-on-insulator (SOI) wafers using a 3-mask process. The brief cross-sectional view of the process flow is shown in Figure S1. The SOI wafer is composed of a 60µm thick heavily doped (100) single crystalline silicon (SCS) device layer with a 4µm thick buried oxide (BOX). After stripping off the native oxide on the device layer surface, 1µm aluminum nitride (AlN) is directly sputtered on SCS, followed by 300nm in-situ doped polycrystalline silicon (poly-Si) by low-pressure chemical vapor deposition (LPCVD). The poly-Si is annealed at 1100°C to activate the dopant. Tetraethyl orthosilicate silicon oxide (TEOS SiO_2) is deposited as a hard mask for dry etching of both the poly-Si and AlN layers. Copper-aluminum alloy (Cu-Al) was sputtered and patterned as contact pads. The last mask defines the resonator geometry and frontside release holes. Deep-RIE (DRIE) is used to etch through the SCS device layer. Finally, the device is released in vapor hydrofluoric acid.

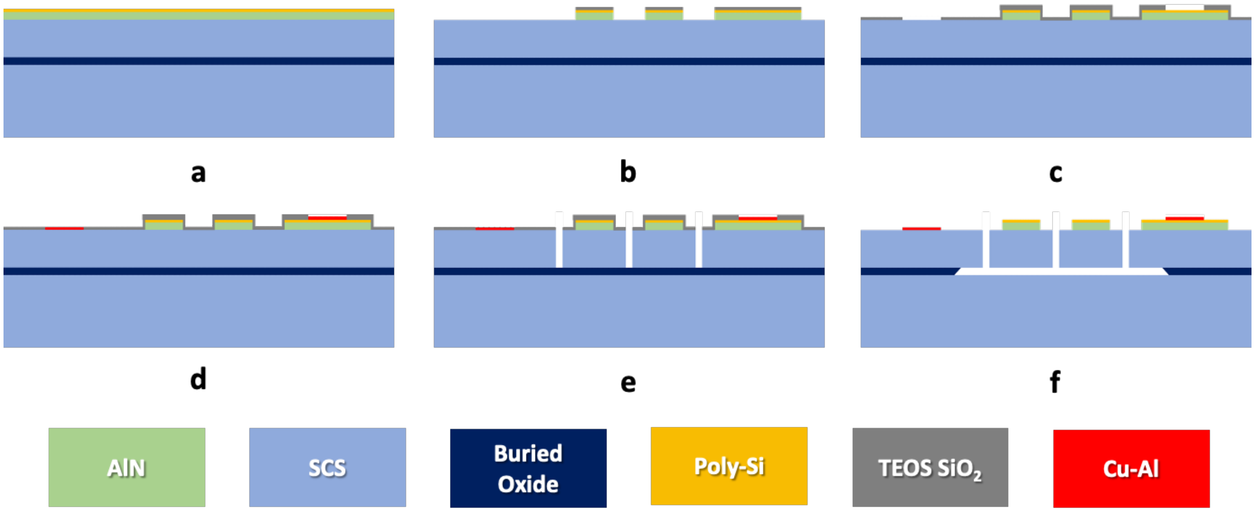

Figure S1: Process flow for fabricating AlN-on-Si resonators: (a) Sputter AlN on SOI substrate and deposit in situ doped LPCVD poly-Si (b) Deposit and pattern TEOS SiO2 as hard mask to etch through top poly-Si and AlN (c) Deposit another TEOS SiO_2 hard mask and pattern vias (d) Sputter and pattern Al-Cu (e) pattern and etch through Si device layer (j) Release the device in vapor HF.

The scanning electron microscope (SEM) photo of a fabricated device is shown in Figure S2.a. The resonator is a side-supported annulus supported by symmetric double T decoupling networks. The center is isolated from the resonator by the SCS DRIE trench. The release holes are defined at the device layer uniformly distributed on the resonator.

Figure S2.b shows a close-up view of the decoupling networks, which guide the electrode traces from the resonator active areas to the peripheral wire-bonding pads. Figure S2.c shows the close-up view of the poly-Si and AlN piezoelectric stack layers. The SCS device layer act as bottom electrode for the ground signal. The etching profile for each layer can also be seen in Figure S2.c.

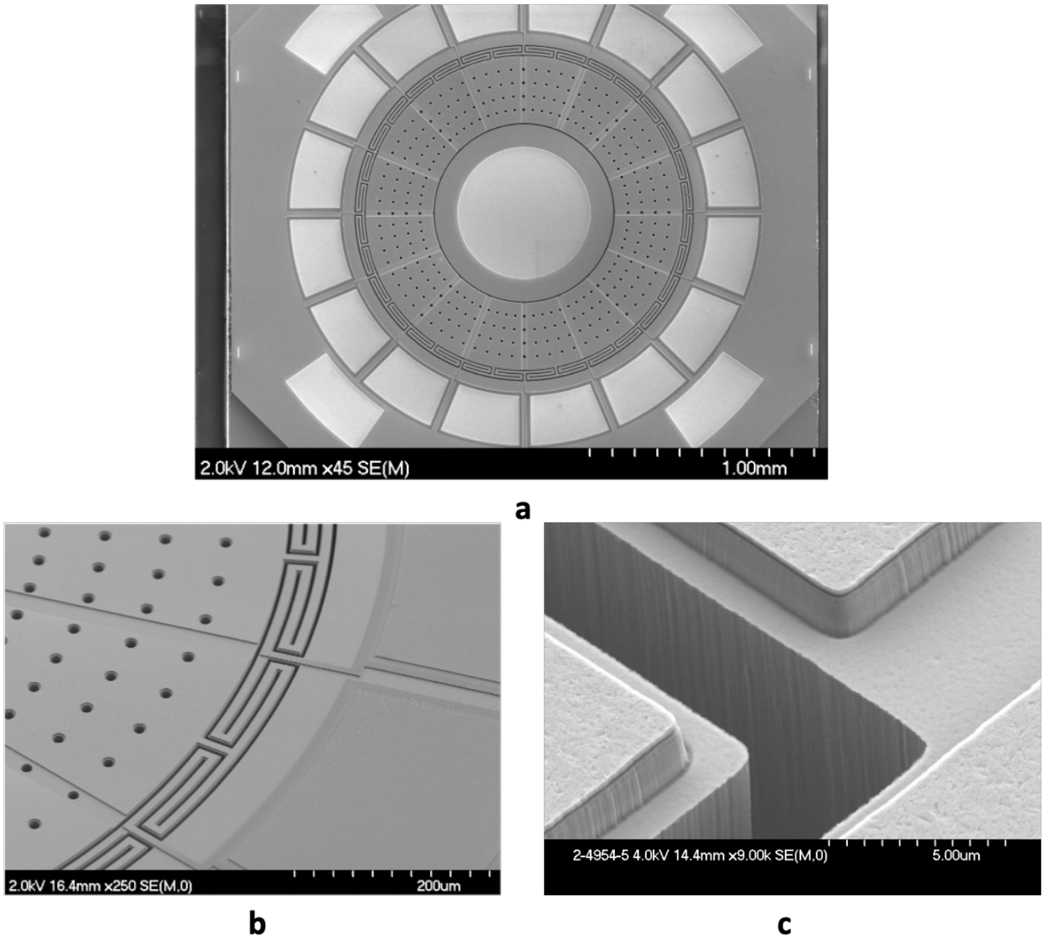

Figure S2: Detailed SEM views of a fabricated device (a) Top view of an AlN-on Si annulus resonator with frontside release holes and side-supported decoupling network (b) The routing of the electrode from the device to the wire-bonding pad through the decoupling network (c) The piezoelectric stack is composed of 300nm poly-Si top electrode and 1µm AlN film on top of SCS device layer.
